# Music Therapy and Other Music-Based Interventions in Pediatric Health Care: An Overview

**DOI:** 10.3390/medicines6010025

**Published:** 2019-02-14

**Authors:** Thomas Stegemann, Monika Geretsegger, Eva Phan Quoc, Hannah Riedl, Monika Smetana

**Affiliations:** 1 Department of Music Therapy, University of Music and Performing Arts Vienna, 1030 Vienna, Austria; smetana-m@mdw.ac.at; 2 WZMF—Music Therapy Research Centre Vienna, University of Music and Performing Arts Vienna, 1010 Vienna, Austria; phan-quoc@mdw.ac.at (E.P.Q.); riedl@mdw.ac.at (H.R.); 3 GAMUT—The Grieg Academy Music Therapy Research Centre, NORCE, 5008 Bergen, Norway; moge@norceresearch.no

**Keywords:** music therapy, music medicine, music-based intervention, pediatrics, developmental medicine, pediatric neurology

## Abstract

**Background:** In pediatric health care, non-pharmacological interventions such as music therapy have promising potential to complement traditional medical treatment options in order to facilitate recovery and well-being. Music therapy and other music-based interventions are increasingly applied in the clinical treatment of children and adolescents in many countries world-wide. The purpose of this overview is to examine the evidence regarding the effectiveness of music therapy and other music-based interventions as applied in pediatric health care. **Methods:** Surveying recent literature and summarizing findings from systematic reviews, this overview covers selected fields of application in pediatric health care (autism spectrum disorder; disability; epilepsy; mental health; neonatal care; neurorehabilitation; pain, anxiety and stress in medical procedures; pediatric oncology and palliative care) and discusses the effectiveness of music interventions in these areas. **Results:** Findings show that there is a growing body of evidence regarding the beneficial effects of music therapy, music medicine, and other music-based interventions for children and adolescents, although more rigorous research is still needed. The highest quality of evidence for the positive effects of music therapy is available in the fields of autism spectrum disorder and neonatal care. **Conclusions:** Music therapy can be considered a safe and generally well-accepted intervention in pediatric health care to alleviate symptoms and improve quality of life. As an individualized intervention that is typically provided in a person-centered way, music therapy is usually easy to implement into clinical practices. However, it is important to note that to exploit the potential of music therapy in an optimal way, specialized academic and clinical training and careful selection of intervention techniques to fit the needs of the client are essential.

## 1. Introduction

Music therapy is an evidence and art-based health profession which uses music experiences within a therapeutic relationship to address clients’ physical, emotional, cognitive, and social needs [1]. A recent worldwide survey among professional members of organizations affiliated with the World Federation of Music Therapy (*n* = 2495) revealed that music therapists mainly worked in mental health settings, schools, geriatric facilities, and private practice [2]. About half of the respondents reported working with children/preteens (50.6%), and teens (45.7%), whereas 38.2% indicated working with infants/children. In the ranking of specific populations served, autism spectrum disorder, developmental disabilities, and depressive disorder are amongst the top three. Although music therapy with children and adolescents constitutes a huge and important part of music therapy practice since the beginnings of the profession, there is a dearth of scientific evidence—particularly when compared to music therapy with adults—and more rigorous research is needed.

The purpose of this overview is to examine the evidence regarding the effectiveness of music therapy and other music-based interventions as applied in pediatric health care. 

### 1.1. Definitions

**Figure 1:**
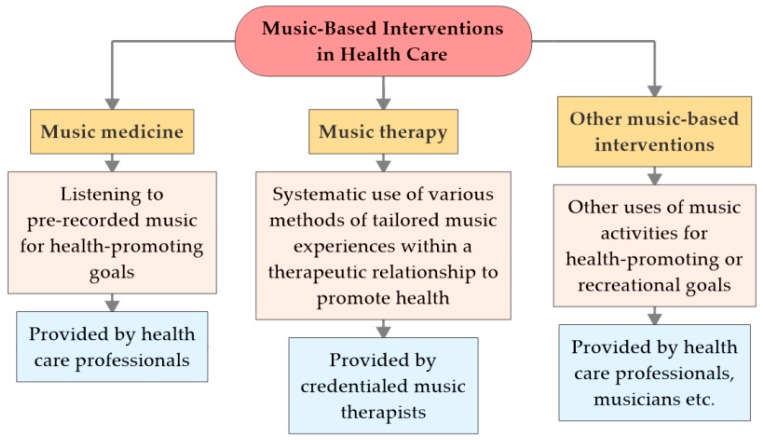
Types of music-based interventions in health care.

This overview defines and contrasts three music-based approaches used in health care: music medicine, music therapy, and other music-based interventions (see Figure 1).

In addition to the definition by the American Music Therapy Association (see above), music therapy (MT) can be described as “a systematic process of intervention wherein the therapist helps the client to promote health, using music experiences and the relationships that develop through them as dynamic forces of change” [3]. With these tailored music experiences provided by credentialed music therapists, music therapy can be contrasted to interventions which are “categorized as ’music medicine’ when passive listening to pre-recorded music is offered by medical personnel” [4], especially before, during and/or after medical interventions, and other music-based interventions such as musically-based activities like choir singing or playing drums that are provided by musicians or health professionals other than credentialed music therapists. 

In MT, four main methods are usually distinguished which overlap in clinical practice or may be combined: improvising, listening, recreating, and composing [5]. Depending on the underlying MT model (see Section 1.2.), the spontaneous creation of music by means of the voice, body, or simple musical instruments may be seen as the ‘via regia’ to the unconscious and may facilitate contact, communication, and emotional expression. Receptive methods (listening to music and responding verbally or in another modality) typically aim to activate or relax a client, to evoke specific body responses, memories, and fantasies, or to stimulate self-knowledge and reflection. Recreating methods encompasses any kind of pre-composed music that the client learns to play or sing. Composition means that the therapist helps the client to create (and to record or perform) music such as instrumental pieces, lyrics, and songs.

### 1.2. Fields of Application and Music Therapy Approaches

The first documentation of MT in children and adolescents comes from shortly after the Second World War, when pioneers in the USA, and from the late 1950s onwards also in Europe, started to use music for treating mentally ill people within various clinical fields [6].

Apart from various clinical areas (see Section 3), MT for children and adolescents is currently also applied in other health care fields such as chronic illness, as well as in non-medical and community contexts such as schools (special education, prevention) or refugee centers (integration, migration, trauma).

Music therapy is especially indicated when verbal language is not, or only limitedly, available or when music as a non-verbal medium enables access to one’s own feelings in cases where improved processing of emotions may help to decrease symptoms. Music therapy can also help to regulate activity and tension and positively influence mood and motivation. In music interventions, it is not necessary for clients to have any musical background such as musical talent, the ability to play an instrument, or to read music; it is one’s individual engagement with the music experience which is a key factor. 

Functional and behavioristic approaches typically use the activating or relaxing effects of music for stimulation or calming, and to enhance learning of specific skills and behaviors. Humanistic approaches (represented by pioneers such as Juliette Alvin [7], or Paul Nordoff and Clive Robbins [8]) emphasize creativity and expression of the self within improvisational music making and the development of positive relationships by allowing the child to find his or her own musical way without fixed rules. Analytically-oriented MT (pioneered by Mary Priestley [9]) employs the symbolic content of improvised music in order to connect with emotions, thoughts, images, or bodily sensations that cannot be verbalized.

In addition to individual and group therapy settings, family-based approaches have been increasingly used in MT with children and adolescents within the last couple of years [10].

## 2. Methods

To evaluate the current evidence for the effectiveness of MT, music medicine, and other music-based interventions in selected fields of pediatric health care, we conducted database searches for systematic reviews published within the last five years (November 2013 to October 2018) using PubMed/Medline, Cinahl, PsycINFO, Scopus, and Web of Science. The following search terms were used: (1) music therapy/music intervention/music-based intervention or arts-based therapy combined with (2) children/pediatrics and with (3) respective fields of application as listed in Section 3 of this article. Based on screening of titles and abstracts, we retrieved eligible systematic reviews. We included those systematic reviews where full-texts were available in English. Findings from systematic reviews and meta-analyses are briefly presented along with a descriptions of assumed working mechanisms and specific goals of music interventions in the Results section.

Included articles were assessed regarding their quality and validity using AMSTAR 2 (A MeaSurement Tool to Assess systematic Reviews) guidelines [11]. AMSTAR 2 is a critical appraisal tool for systematic reviews that include randomized or non-randomized studies of healthcare interventions, or both. It includes 16 items on domains such as “adequacy of the literature search”, “justification for excluding individual studies”, “appropriateness of meta-analytical methods”, and “consideration of risk of bias when interpreting the results of the review”. Based on a scheme for interpreting weaknesses detected in critical and non-critical items, the overall confidence in the results of the review can then be categorized as “high”, “moderate”, “low”, or “critically low”. All of the systematic reviews and meta-analyses included in our overview were assessed and rated independently by two of the authors. Any disagreements were discussed further in order to reach mutual consent between the two authors. 

## 3. Results

We included a total of 13 systematic reviews/meta-analyses—published within the last five years—across the following fields of pediatric health care (in alphabetical order; in parentheses; number of systematic reviews included): autism spectrum disorder (2); disability (1); epilepsy (1); mental health (2); neonatal care (3); neurorehabilitation (1); pain, anxiety, and stress in medical procedures (2); pediatric oncology and palliative care (1). Key characteristics of the studies and an assessment of quality according to the AMSTAR 2 guidelines are summarized in Table 1.

### 3.1. Autism Spectrum Disorders

Music therapy has been applied in the field of autism since the mid-1940s [12]. The latest update of a Cochrane review on music therapy for people with autism spectrum disorders (ASD) [13] summarizes results from 10 studies examining the short- and medium-term effect of MT interventions (one week to seven months) with a total of 165 participants who were all between two and nine years of age. Findings provide evidence for moderate to large effects of MT as compared to ‘placebo’ therapy or standard care in outcome areas constituting the core of the condition such as social interaction, non-verbal communicative skills, initiating behavior, and social–emotional reciprocity. Music therapy may also help to improve verbal communication, social adaptation, joy, and the quality of parent–child relationships. Due to low numbers of study participants and other study design issues, the quality of the evidence was assessed as moderate to low. Therefore, the review authors suggest that future studies need to be larger, more rigorous, and should also evaluate outcomes for people with ASD above nine years of age. Based on AMSTAR 2 criteria, the confidence in these results can be assessed as “high” (i.e., the SR provides an accurate and comprehensive summary of the results of the available studies).

Another meta-analysis that focused on randomized controlled trials (RCTs) published in Chinese [14] also came to favorable conclusions regarding MT for ASD, but only reached a rating of “low” confidence according to AMSTAR 2 (i.e., it has a critical methodological flaw and may not provide an accurate and comprehensive summary of the available studies). Summarizing the findings of six studies with a total of 300 children with autism, the authors found significant effects of MT (six weeks to three months) as compared to other forms of therapy on mood, language, sensory perception, behavior, and social skills. The risk of bias for all six included studies was assessed as moderate. 

The potential of MT as an intervention within ASD, where difficulties with social interaction and communication are at the core by definition, is explained by processes that naturally occur in musical interactions within the relationship between client and therapist, where music is used as an expressive and communicative means. Behaviors necessary for social engagement such as joint attention, eye contact, and turn-taking are characteristic events in shared, active music making and therefore inherent components of MT processes. Structures in improvised or pre-composed music also provide opportunities to experience both predictability and flexibility, and attention and enjoyment typically increase in individuals when presented with musical as opposed to verbal stimuli. Music therapy for individuals with ASD is often provided as individual therapy, but there are also group-based, peer-mediated and family-based forms [13]. 

### 3.2. Disability

In MT, work with children and adolescents with disabilities is one of the traditional fields of application [6]. Children with ASD, trisomy 21, Rett syndrome, or Williams syndrome are known to be very responsive to music listening and musical activities. Thus, MT is applied for assessment as well as for fostering communication, social competencies, emotional regulation, and motor skills [15,16]. Although MT is a quite common approach in special education, there is still a dearth of research, in particular with respect to effectiveness studies. 

Only one SR met our inclusion criteria [17]. This publication on “music research in inclusive school settings” covered the period from 1975 to 2013 and found evidence that music-based interventions in preschool settings positively influence reading/literacy outcomes in children with and without disabilities. Due to the high level of heterogeneity in study methodologies and outcomes, no other summary statements could be made. As a conclusion, the authors stress the necessity to conduct more studies in inclusive music settings. Due to several critical flaws in the review, the confidence in its results was rated as “critically low” according to AMSTAR 2 criteria, which means that it should not be relied upon to provide an accurate and comprehensive summary of the included studies.

### 3.3. Epilepsy

Musicogenic epilepsy, i.e. epileptic seizures induced by music, has been known of since at least the late 1930s, as Oliver Sacks mentions in his book “Musicophilia” [18]. At the same time, music has the potential to reduce seizure activity: “The dichotomous effect of music on seizures may be explained by modification of dopaminergic circuitry or counteractive cognitive and sensory input in ictogenesis” [19]. In a recent systematic review [20], eight publications were identified in which the influence of music by W.A. Mozart on seizures in children was studied. Although there is some substantial and serious doubt about the existence of a ‘Mozart effect’ as such, classical pieces of the ‘Wunderkind’ are still very popular stimuli in this type of research. Noteworthily, seven of the eight included studies were from the same research group in Taiwan. Brackney and Brooks [20] summarize their findings: “The evidence for the efficacy of the Mozart Effect on seizure activity in children is promising but not conclusive”. According to AMSTAR 2 criteria, the confidence in the systematic review’s results is rated as “critically low”. Seven of the studies were classified as “quasi-experimental”. In the only RCT [21], the treatment group (*n* = 24) listened to Mozart’s sonata for two pianos in D major K.448 daily before bedtime for six months, while the control group (*n* = 24) received treatment as usual (patients were between 8 and 13 years of age). Results showed that during the follow-up period of approximately one and a half years on average, eight of the 22 patients in the treatment group suffered seizure recurrence, while 18 of the 24 patients in the control group had seizure recurrence. Further, significant decreases in epileptiform discharges after one, two, and six months compared with EEGs before listening to music have been observed in the treatment group.

### 3.4. Mental Health

Mental health care for children and adolescents is one of the main clinical fields of music therapists. Music therapy with children and adolescents can include active methods such as improvisation or working with songs (song writing, performing of pre-composed songs) as well as receptive methods such as listening to pre-recorded music [5].

A Cochrane review on music therapy for depression [22]—for which the AMSTAR 2 level of confidence in the results was rated as “high”—included nine RCTs (total *n* = 421), of which two studied the effectiveness of “music therapy techniques” in high school students [23,24]. However, as it was not clear whether a trained music therapist was providing the interventions [22], these studies are categorized as music-based intervention studies according to our definitions provided above. Findings from these two studies suggest that the group music intervention in comparison to cognitive behavioral therapy is significantly more effective as measured by self-rating (Beck Depression Inventory).

The findings of a recent meta-analysis [25] on different music-based interventions (including MT, music medicine, and other music-based interventions) to reduce internalizing symptoms in children and adolescents also suggest that these interventions are beneficial, but due to a relatively small sample size (only five trials with a combined sample size of *n* = 100), the authors draw these implications with caution. The confidence in the results according to AMSTAR 2 criteria was estimated as “low”.

### 3.5. Neonatal Care

Music therapy and music interventions are of growing importance in neonatal intensive care units (NICUs) as documented by two recent publications: a systematic review of RCTs on various music-based interventions by van der Heijden and colleagues [26], and a meta-analysis on music therapy for infants and their parents by Bieleninik, Ghetti, and Gold [27]. Progress in medicine and new technical developments allow for higher survival rates in preterm newborns. However, the survival of preterm babies who are more premature and vulnerable also calls for better and more efficient integrated care as early as possible. This explains why there is a growing awareness for environmental factors influencing the newborn’s health and well-being, e.g. acoustic stimuli in the NICU. Van der Heijden and colleagues state: “Where unpredictable noise adversely affects sleep and physiologic stability, meaningful auditory stimulation, such as music, might contribute to the neurodevelopment of premature infants” [26].

Summarising the main findings of the two reviews—based on RCTs only, and both assessed as justifying “moderate” confidence in their findings according to AMSTAR 2 (i.e., they include weaknesses, but no critical flaws, so that they may provide an accurate summary of the results of included studies)—MT and other music-based interventions in NICUs lead to a reduction in heart and respiratory rate, improve the infant’s sleep and food intake, and reduce the anxiety of mothers [26,27]. Interestingly, not only from an economical point of view, a recent systematic review of RCTs [28] found that length of stay can be significantly reduced through music therapy interventions. In addition, O’Toole et al. [28] reported that music medicine interventions yield positive effects of pain management in preterm infants. However, the confidence in this review’s results had to be rated as “critically low” according to AMSTAR 2 criteria due to several critical flaws in its methodology.

Regardless of whether live or pre-recorded music is played, the ‘golden rules’ of music interventions in the NICU are “less is more”, and “minimal change, minimal range”. The former being true for duration and the number of musical instruments used, the latter applies to all musical parameters: “minimal change” in rhythm, harmony, dynamics, and volume, and “minimal range” in melody and pitch range—“like a lullaby” [29]. Thus, in live interventions, music therapists primarily use their voice (infant directed singing), accompanied maybe by a harp, a guitar or a small percussion instrument. For recorded acoustic interventions, music or the mother’s voice is played softly through loudspeakers inside or outside of the incubator.

### 3.6. Neurorehabilitation

A recent Cochrane review on MT for acquired brain injury came to the following conclusion: “The results suggest that music interventions using rhythm may be beneficial for improving walking in people with stroke, and this may improve quality of life. (…) Music interventions that use a strong beat within music may be more effective than interventions where a strong beat is used without music. Treatment delivered by a trained music therapist might be more effective than treatment delivered by other professionals” [30]. The quality of the evidence was assessed as “generally low” by the review authors [30]. The confidence in these results can be assessed as “high” based on AMSTAR 2 criteria. In the context of our focus on pediatric health care, it has to be noted however that it was not possible to determine the number of adolescents who were included based on the information given in the review. According to the selection criteria of the review, “studies that included people older than 16 years of age” were examined. Most of the studies report a mean age of more than 50 years of age, so the applicability of the review’s results to children and adolescents remains unclear.

### 3.7. Pain, Axiety, and Stress in Medical Procedures

A systematic review by Yinger and Gooding [31] on music-based interventions for procedural support identified 50 studies meeting the inclusion criteria, but only eight of them included children and adolescents. The confidence in the results of this systematic review according to AMSTAR 2 criteria was rated as “moderate”, i.e., it includes weaknesses, but no critical flaws, so that it may provide an accurate summary of the results of included studies. The authors came to the conclusion that the majority of studies (84%) were at high risk of bias and revealed limitations in adequate intervention reporting. Interestingly, two of the eight studies with a low or moderate risk of bias were music therapy studies involving pediatric participants [32,33], with significant effects for the reduction of pain and anxiety. 

In a systematic review from 2016, Kim and Stegemann [34] searched the literature of the last 35 years with regard to music listening as an intervention for children and adolescents. The authors identified 36 studies of which 18 were from the field of pediatrics, encompassing 12 studies with pediatric patients undergoing either surgery or needle insertion procedures. Accordingly, pain, anxiety, and stress were the main outcome measures.

Pain perception in the context of medical procedures was investigated in 12 RCTs, of which nine found a significant decrease of pain in the music condition compared to the non-music condition or treatment as usual. In most of the studies, the music condition included recorded music (e.g., lullabies, classical music, pop) presented via loudspeaker or earphones. The largest effect sizes were reported in a study by Nguyen and colleagues [35] who investigated the reduction of pain and anxiety in children with cancer undergoing lumbar puncture (LP). Pain, heart and respiratory rates were significantly reduced in the music group during and after the LP (pain reduction: *d* = 1.53 (huge effect) during and *d* = 1.08 (large effect) after the LP).

Besides pain, anxiety plays a major role as a stressor for children in medical procedures. The effect of music listening in reducing anxiety was measured in 11 studies, of which seven favored the music condition while four studies found no significant difference between groups. Effect sizes for anxiety reduction ranged between *d* = 0.61 (medium effect) and *d* = 1.5 (huge effect). Kristjánsdóttir and Kristjánsdóttir [36] studied the effect of a specific music medicine intervention (a musical distraction strategy) in adolescents receiving immunization. They found the odds of participants experiencing “no pain” during the immunization if listening to music to be approximately 2.8 times higher than those of participants receiving standard nursing care. The authors concluded that musical distraction, pre-immunization fear and anxiety, and expected immunization pain were significant predictors of adolescent immunization pain sensation.

The effects of music listening on stress perceived by children and adolescents during painful medical procedures were measured by observational parameters (e.g., video analysis) as well as physiological parameters (e.g., heart rate, blood pressure, respiratory rate). The majority of the studies (four out of seven) were in favor of the music condition, while the other three studies found no significant differences. Results of an earlier RCT by Malone [37] who used live music interventions with children in a preoperative setting revealed that participants in the music condition showed significantly shorter duration of stress signs with a large effect size (*d* = 1.01). 

Only two of the 36 studies reviewed by Kim and Stegemann [30] were categorized as “relatively low risk of bias”; both of these studies [35,38] showed strong results in favor of the music medicine intervention. The confidence in the results of this systematic review by Kim and Stegemann [30] according to AMSTAR 2 criteria was also rated as “moderate”.

### 3.8. Pediatric Oncology and Palliative Care

In several countries, music therapy services are well-established in the field of pediatric oncology, and some treatment guidelines include creative arts therapies for this specific client population, as for instance in Germany [39]. Music therapists in pediatric oncology and palliative care have to deal with various somatic and psychological symptoms of their patients and often, therapy is provided for children together with their family members. Due to ethical concerns and feasibility issues regarding such vulnerable times in life, RCTs are scarce in this field, particularly within palliative care.

A Cochrane review by Bradt and colleagues [40]—for which the AMSTAR 2 level of confidence in the results was assessed as “high”—on music interventions for improving psychological and physical outcomes in cancer patients included studies with children, but only five of the 52 reviewed studies were conducted in pediatric fields. Outcomes of these studies varied from impact on immune system functioning [41] through to anxiety and pain management [35,42] and children´s coping behavior [43,44]. Due to the low number of studies in pediatrics, no overall conclusions were drawn. Findings from single studies indicate some benefits of MT and music-based interventions, particularly on anxiety, pain, and coping behaviors.

## 4. Discussion

According to the results from systematic reviews and meta-analyses, the evidence for the effectiveness of music therapy and other music-based interventions in areas relevant to pediatric health care can be summarized as displayed in Table 2.

Music therapy (MT) and other music-based interventions are applied and have shown to be beneficial in a broad variety of fields and seem effective especially in combination with other treatment forms and within a multimodal therapy approach—but they are certainly not the ‘magic bullet’ working for everyone at any time. 

The growing body of evidence for MT and other music-based interventions (including music medicine) in childhood and adolescence indicates that MT is particularly effective in improving mood and affect regulation, communication, social skills, and quality of life; music medicine approaches are successfully applied in medical settings to alleviate pain, anxiety, and stress. As documented by meta-analyses, the best evidence regarding the effectiveness of MT today is reported in neonatal care and in children with autism spectrum disorders. In other fields, especially in children with disabilities, there is a clear need for more and better-quality research—which is of course not only a challenge for MT but holds true for medical and special education interventions in childhood and adolescence in general. 

“Where words fail, music speaks”, as the writer Hans Christian Andersen put it. Thus, music-based interventions can open doors, especially for people who are not capable of communicating through spoken language. The communication beyond words is a unique feature of arts therapies such as MT—this may be one reason why MT works in NICUs and for people with ASD. 

Music therapy can be considered a safe and generally well-accepted intervention in pediatric health care to alleviate symptoms and improve quality of life. None of the included systematic reviews reported adverse effects of music-based interventions for children and adolescents. This is in line with the findings of a study on the acceptance of specific complementary and alternative medicine modalities, where acceptance was highest for music therapy [45].

As an individualized intervention that is typically provided in a person-centered way, music therapy is usually easy to implement into clinical practices. In addition, it is important to note that to exploit the potential of music therapy in an optimal way, specialized academic and clinical training and careful selection of intervention techniques to fit the client’s needs are essential. More rigorous research on MT, music medicine, and other music-based interventions is still needed to determine what types of interventions work best for whom and under which circumstances.

## Figures and Tables

**Table 1:** Key characteristics and ratings of overall confidence in the results (based on AMSTAR 2) of included systematic reviews.

Field of Application	Author(s), Year (Type(s) of Intervention Studied) *	Number of Studies/Participants (Total)	(Primary) Outcomes	Confidence in the Results
**Autism Spectrum Disorders**	Geretsegger et al., 2014 [13] [MT]	10/165	Social interaction; non-verbal and verbal communicative skills; initiating behavior; social–emotional reciprocity	High
	Shi et al., 2016 [14] (MT)	6/300	Mood; language; behavior; sensory perception; social skills	Low
**Disability**	Jellison and Draper, 2015 [17] (MT, MBI)	22/> 562 ^1^	Behavior in the categories: music, social, academic, motor, on-task	Critically low
**Epilepsy**	Brackney and Brooks, 2018 [20] (MM, MBI)	8/268	Seizure frequency; epileptiform activity (EEG)	Critically low
**Mental Health**	Aalbers et al., 2017 [22] (MT, MBI)	9/421 ^2^	Clinician-rated and patient-reported depressive symptoms	High
	Geipel et al., 2017 [25] (MT, MM, MBI)	5/195	Internalizing symptoms	Low
**Neonatal Care**	van der Heijden et al., 2016 [26] (MT, MM, MBI)	20/1128	Physiological parameters; growth and feeding; behavioral state; relaxation Outcomes and pain	Moderate
	Bieleninik et al., 2016 [27] (MT)	16/1071 ^3^	Physiological and behavioral parameters; maternal anxiety; service-level outcome	Moderate
	O’Toole, 2017 [28] (MT, MM, MBI)	12/918	Physiological indicators; feeding behaviors; length of stay; pain	Critically low
**Neuro-rehabilitation**	Magee et al., 2017 [30] (MT, MM, MBI)	29 ^4^/775	Gait, upper extremity function	High
**Pain, Anxiety and Stress in Medical Procedures**	Yinger and Gooding, 2015 [31] (MT, MM)	50 ^5^/4379	Pain and anxiety during medical procedures	Moderate
	Kim and Stegemann, 2016 [34] (MT, MM)	36/1990	Three categories: pediatrics (e.g., pain, anxiety, stress); mental health; miscellaneous	Moderate
**Pediatric Oncology and Palliative Care**	Bradt et al., 2016 [40] (MT, MM)	52 ^6^/3731	Psychological outcomes (e.g., depression, anxiety); physical outcomes (e.g., fatigue, nausea, pain)	High

* MT = music therapy; MM = music medicine; MBI = other music-based interventions. ^1^ In some of the included studies, the numbers of participants were not indicated or unclear. ^2^ In two of the nine publications [35,36], adolescent patients were studied (total *n* = 82). ^3^ Number of infant participants; in addition, 286 parent participants were included. ^4^ According to the selection criteria of the review, “studies that included people older than 16 years of age” were examined; based on the information given in the review it is not possible to indicate how many adolescents were included; most of the studies report a mean age > 50 years of age. ^5^ Eight of the 50 studies involved pediatric participants (total *n* = 705). ^6^ Five of the 52 publications were conducted in pediatric patients (total *n* = 201).

**Table 2:** Summary of findings regarding evidence for the effectiveness of music therapy (MT), music medicine (MM) and other music-based interventions (MBI) in selected fields of applications relevant to pediatric health care.

Field of Application	Findings
**Autism Spectrum Disorders**	MT can improve social interactions, non-verbal communicative skills, initiating behavior, and social–emotional reciprocity (moderate to strong effects) [13]
	MT can improve verbal communication, social adaptation, joy, and the quality of parent–child relationships [13]
	MT can improve mood, language, sensory perception, behavior, and social skills [14]
**Disabilities**	MBI can positively influence reading/literacy outcomes in children with and without disabilities [17]
**Epilepsy**	MBI can reduce epileptiform discharges [20]
	Evidence for the efficacy of music medicine interventions using the Mozart Effect on seizure activity in children is promising but not conclusive [20]
**Mental Health**	MBI in an educational setting can have an anti-depressive effect in adolescents [22]
	MT/MM/MBI can reduce internalizing symptoms [25]
**Neonatal Care**	MT/MM can decrease heart and respiratory rate [26,27]
	MT can improve infant sleep [26,27] and food intake [26,27,28]
	MT/MM can reduce the anxiety of mothers [26,27]
	MT can reduce the length of stay in hospital [28]
	MM can reduce pain during blood-drawing procedures [28]
**Neurorehabilitation**	Effects of MT/MM/MBI on adolescents are unclear due to missing differentiation/data for the age group 16–18 years [30]
**Pain, Anxiety and Stress in Medical Procedures**	MT/MM can decrease pain [31]
	MT/MM can reduce anxiety [31,34]
	MT/MM can decrease levels of stress parameters (heart and respiratory rate) [34]
**Pediatric Oncology and Palliative Care**	MT can improve pain management [40]
	MT can improve children’s coping behavior [40]
	MT can improve immunological status (increase of IgA level) [40]

## References

[1] American Music Therapy Association (2018). What Is Music Therapy. https://www.musictherapy.org/about/musictherapy/.

[2] Kern P., Tague D.B. (2017). Music therapy practice status and trends worldwide: An international survey study. J. Music Ther..

[3] Bruscia K.E. (1998). Defining Music Therapy.

[4] Bradt J., Dileo C., Shim M. (2013). Music interventions for preoperative anxiety. Cochrane Database Syst. Rev..

[5] Stegemann T., Geretsegger M., Thompson B. (2014). Music therapy methods. Music in the Social and Behavioral Sciences.

[6] Davis W.B., Gfeller K.E., Davis W.B., Gfeller K.E., Thaut M.H. (2008). Music therapy: Historical perspective. Introduction to Music Therapy: Theory and Practice.

[7] Alvin J. (1965). Music for the Handicapped Child.

[8] Nordoff P., Robbins C. (1977). Creative Music Therapy: Individualized Treatment for the Handicapped Child.

[9] Priestley M. (1975). Music Therapy in Action.

[10] Jacobsen S.L., Thompson G. (2017). Music Therapy with Families. Therapeutic Approaches and Theoretical Perspectives.

[11] Shea B.J., Reeves B.C., Wells G.A., Micere T., Candyce H., Julian M., Moher D., Tugwell P., Welch V., Kristjansson E. (2017). AMSTAR 2: A critical appraisal tool for systematic reviews that include randomised or non-randomised studies of healthcare interventions, or both. BMJ.

[12] Reschke-Hernández A.E. (2011). History of music therapy treatment interventions for children with autism. J. Music Ther..

[13] Geretsegger M., Elefant C., Mössler K.A., Gold C. (2014). Music therapy for people with autism spectrum disorder. Cochrane Database Syst. Rev..

[14] Shi Z.M., Lin G.H., Xie Q. (2016). Effects of music therapy on mood, language, behavior, and social skills in children with autism: A meta-analysis. Chin. Nurs. Res..

[15] Hintz M. (2013). Guidelines for Music Therapy Practice in Developmental Health.

[16] Lathom-Radocy W.B. (2014). Pediatric Music Therapy.

[17] Jellison J.A., Draper E.A. (2015). Music research in inclusive school settings: 1975 to 2013. J. Res. Music Educ..

[18] Sacks O. (2007). Musicophilia. Tales of Music and the Brain.

[19] Maguire M. (2015). Music and its association with epileptic disorders. Prog. Brain Res..

[20] Brackney D.E., Brooks J.L. (2018). Complementary and alternative medicine: The Mozart Effect on childhood epilepsy—A systematic review. J. Sch. Nurs..

[21] Lin L.C., Lee M.W., Wei R.C., Mok H.K., Yang R.C. (2014). Mozart K.448 listening decreased seizure recurrence and epileptiform discharges in children with first unprovoked seizures: A randomized controlled study. BMC Complement. Altern. Med..

[22] Aalbers S., Fusar-Poli L., Freeman R.E., Spreen M., Ket J.C.F., Vink A.C., Maratos A., Crawford M., Chen X.J., Gold C. (2017). Music therapy for depression. Cochrane Database Syst. Rev..

[23] Hendricks C.B., Robinson B., Bradley B., Davis K. (1999). Using music techniques to treat adolescent depression. J. Humanist. Couns. Educ. Dev..

[24] Hendricks C.B. (2001). A study of the use of music therapy techniques in a group for the treatment of adolescent depression. Diss. Abstr. Int..

[25] Geipel J., Koenig J., Hillecke T.K., Resch F., Kaess M. (2018). Music-based interventions to reduce internalizing symptoms in children and adolescents: A meta-analysis. J. Affect. Disord..

[26] Van der Heijden M.J.E., Oliai Araghi S., Jeekel J., Reiss I.K.M., Hunink M.G.M., van Dijk M. (2016). Do hospitalized premature infants benefit from music interventions? A systematic review of randomized controlled trials. PLoS ONE.

[27] Bieleninik Ł., Ghetti C., Gold C. (2016). Music therapy for preterm infants and their parents: A meta-analysis. Pediatrics.

[28] O’Toole A., Francis K., Pugsley L. (2017). Does music positively impact preterm infant outcomes?. Adv. Neonatal Care.

[29] Nöcker-Ribaupierre M., Bradt J. (2013). Premature Infants. Guidelines for Music Therapy Practice in Pediatric Care.

[30] Magee W.L., Clark I., Tamplin J., Bradt J. (2017). Music interventions for acquired brain injury. Cochrane Database Syst. Rev..

[31] Yinger O.S., Gooding L.F. (2015). A systematic review of music-based interventions for procedural support. J. Music Ther..

[32] Noguchi L.K. (2006). The effect of music versus nonmusic on behavioral signs of distress and self-report of pain in pediatric injection patients. J. Music Ther..

[33] Whitehead-Pleaux A.M., Baryza M.J., Sheridan R. (2006). The effects of music therapy on pediatric patients’ pain and anxiety during donor site dressing change. J. Music Ther..

[34] Kim J., Stegemann T. (2016). Music listening for children and adolescents in health care contexts: A systematic review. Art Psychother..

[35] Nguyen T.N., Nilsson S., Hellström A.L., Bengtson A. (2010). Music therapy to reduce pain and anxiety in children with cancer undergoing lumbar puncture: A randomized clinical trial. J. Pediatr. Oncol. Nurs..

[36] Kristjánsdóttir Ó., Kristjánsdótti4 G. (2011). Randomized clinical trial of musical distraction with and without head-phones for adolescents’ immunization pain. Scand. J. Caring Sci..

[37] Malone A.B. (1996). The effects of live music on the distress of pediatric patients receiving intravenous starts, venipunctures, injections, and heel sticks. J. Music Ther..

[38] Hartling L., Newton A.S., Liang Y., Jou H., Hewson K., Klassen T.P., Curtis S. (2013). Music to reduce pain and distress in the pediatric emergency department: A randomized clinical trial. JAMA Pediatr..

[39] Schröder H., Lilienthal S., Schreiber-Gollwitzer B., Griessmeier B., Leiss U. Psychosoziale Versorgung in der Pädiatrischen Onkologie und Hämatologie. https://www.awmf.org/uploads/tx_szleitlinien/025-002l_S3_Psychosoz-Versorgung-Paed-Onkol-Haematol-2013-abgelaufen.pdf.

[40] Bradt J., Dileo C., Magill L., Teague A. (2016). Music interventions for improving psychological and physical outcomes in cancer patients. Cochrane Database Syst. Rev..

[41] Duocastella A.C. (1999). Effect of music on children with cancer. Rev. Enferm..

[42] Bufalini A. (2009). Role of interactive music in oncological paediatric patients undergoing painful procedures. Minerva Pediatr..

[43] Burns D.S., Robb S.L., Haase J.E. (2009). Exploring the feasibility of a therapeutic music video intervention in adolescents and young adults during stem-cell transplantation. Cancer Nurs..

[44] Robb S.L., Burns D.S., Stegenga K.A., Haut P.R., Monahan P.O., Meza J., Stump T.E., Cherven B.O., Docherty S.L., Hendricks-Ferguson V.L. (2014). Randomized clinical trial of therapeutic music video intervention for resilience outcomes in adolescents/young adults undergoing hematopoietic stem cell transplant: A report from the Children’s Oncology Group. Cancer.

[45] Trifa M., Tumin D., Walia H., Lemanek K.L., Tobias J.D., Bhalla T. (2018). Caregivers’ knowledge and acceptance of complementary and alternative medicine in a tertiary care pediatric hospital. J. Pain Res..

